# A protocol for a systematic review of clinical practice guidelines for the antenatal management of dichorionic diamniotic twin pregnancy

**DOI:** 10.12688/hrbopenres.13418.1

**Published:** 2021-10-22

**Authors:** Caroline O'Connor, Sara Leitao, Keelin O'Donoghue

**Affiliations:** 1 Infant Centre, University College Cork, Cork, Ireland; 2 Pregnancy Loss Research Group, Department of Obstetrics and Gynaecology, University College Cork, Cork, Ireland; 3 National Perinatal Epidemiology Centre (NPEC), University College Cork, Cork, Ireland

**Keywords:** Dichorionic diamniotic, multiple pregnancy, twin pregnancy, clinical practice guidelines, protocol, antenatal management.

## Abstract

**Overview:** The protocol outlines the process designed to systematically review clinical practice guidelines (CPGs), addressing the antenatal management of dichorionic diamniotic (DCDA) twin pregnancies.

**Background:** CPGs are statements that include recommendations intended to optimise patient care, that are informed by a systematic review of evidence and an assessment of the benefits and harms of alternative care options. CPGs are typically created by scientific institutes, organisations and professional societies, and high-quality CPGs are fundamental to improve patient outcomes, standardise clinical practice and improve the quality of care. While CPGs are designed to improve the quality of care, to achieve this, the identification and appraisal of current international CPGs is required. Because twin pregnancies are identified as high-risk pregnancies, a systematic review of the CPGs in this field is a useful first step for establishing the required high level of care.

**Aim:** The aim of the systematic review is to identify, appraise and examine published CPGs for the antenatal management of DCDA twin pregnancies, within high-income countries.

**Methods:** We will identify published CPGs addressing any aspect of antenatal management of care in DCDA twin pregnancies, appraise the quality of the identified CPGs using the Appraisal of Guidelines for Research and Evaluation version 2 (AGREE II) the Appraisal of Guidelines Research and Evaluation – Recommendations excellence (AGREE-REX) instruments and examining the recommendations from the identified CPGs. Ultimately, this protocol aspires to clearly define the process for a reproducible systematic review of CPGs within a high-income country, addressing any aspect of antenatal management of DCDA twin pregnancies.

**PROSPERO registration:** CRD42021248586 (24/06/2021)

## Introduction

Twin pregnancies are fundamentally defined and managed according to their chorionicity (number of outer membranes in a multiple pregnancy) and amnionicity (number of inner membranes that surround babies in a multiple pregnancy) ^1^. A twin pregnancy can originate from the fertilisation of two oocytes (dizygotic) or from the fertilisation of one oocyte that splits (monozygotic). Dizygotic twin pregnancies will always be dichorionic diamniotic (DCDA) and have previously been referred to as non-identical twins or fraternal twins. A monozygotic twin pregnancy, also referred to as identical twins, can be comprised of dichorionic diamniotic (30%), and monochorionic monoamniotic diamniotic or monochorionic diamniotic (70%); the chorionicity and amnionicity is determined by the time between fertilisation and embryonic cleavage ^2^. Globally monozygotic twins occur in 3.5 per 1000 pregnancies, and this rate remains relatively constant ^3^. Interestingly, rates of dizygotic twins vary according to geographical location, with Nigeria having the highest incidence, 40 of every 1000, and Japan the lowest at 6.7 per 1000 ^4^.

There is global consensus that twin pregnancies are higher risk compared to singleton pregnancies because they increase the risk of morbidity and mortality for the mother and child, and they present unique challenges for both families and health care providers ^5^. Maternal obstetric morbidities are observed more commonly ^6, 7^ and there is an increased perinatal risk of adverse outcomes including preterm birth, intrauterine growth restriction and congenital abnormalities, thus contributing to a higher incidence of perinatal mortality ^8– 11^). The MMBRACE (Mothers and Babies: Reducing Risk through Audits and Confidential Enquiries across the UK) published its report on the Confidential Enquiry into Stillbirth and Neonatal Death in Twin Pregnancies in January 2021 and reported ‘major sub-optimal antenatal care in 50% of pregnancies’ and ‘in around 1 in 2 baby deaths, the care was poor, and that if the care had been better it may have prevented the baby from dying’ ^12^.

Given the associated risk with twin pregnancies and the related adverse outcomes, there is a need for consistent clinical care of twin pregnancies that follows the best evidence-based practice. To safeguard the effectiveness of clinical practice and future research we need to ensure the clinical guidelines are of high quality and gaps are identified as a focus for future guideline development and updates.

Chorionicity is one of the most important factors influencing perinatal outcome in twin pregnancies and is ideally diagnosed in early pregnancy ^13– 15^. Dichorionic twin pregnancies are most common and account for 70% of twin pregnancies, while monochorionic account for 30% ^16^. Diagnosis of placental chorionicity is essential for the management of twin pregnancies, as intertwin vascular anastomoses are present in most monochorionic twin placentas, leading to unique complications ^17^. In recognition of the unique complications associated with monochorionic twin pregnancies specific international guidelines exist to address the management of these pregnancies.

Clinical practice guidelines (CPGs) are “statements that include recommendations intended to optimise patient care that are informed by a systematic review of evidence and an assessment of the benefits and harms of alternative care options” ^18^. CPGs are typically created by scientific institutes, organisations and professional societies, and high-quality CPGs are fundamental to improve patient outcomes, standardise clinical practice and improve the quality of care ^19, 20^. CPGs for the management of twin pregnancies exist at national and international level, however the amount, quality and consistency of these CPGs is currently unknown.

### Aim of this review

The aim of this systematic review is to identify, appraise (using the Appraisal of Guidelines for Research and Evaluation version 2 (AGREE II) and the Appraisal of Guidelines Research and Evaluation – Recommendations excellence (AGREE REX) tools) and examine published clinical practice guidelines (CPGs) for the antenatal management of dichorionic and diamniotic (DCDA) twin pregnancies, within high income countries.

The specific objectives are to:

1. Identify published CPGs addressing any aspect of antenatal management of care in DCDA twin pregnancies within high income countries.
2. Appraise the quality of the identified CPGs using the Appraisal of Guidelines for Research and Evaluation version 2 (AGREE II) the Appraisal of Guidelines Research and Evaluation – Recommendations excellence (AGREE-REX) instruments.
3. Examine the recommendations from the identified CPGs concerning antenatal management of DCDA twin pregnancies.

## Methods

### Protocol

The present protocol has been registered (24/06/2021) in the International Prospective Register of Systematic Reviews database (PROSPERO ID: CRD42021248586) and is reported in accordance with the reporting guidance provided in the Preferred Reporting Items for Systematic review and Meta-Analysis – Protocols (PRISMA-P) 2015 ^21^; the completed checklist is available in *Reporting guidelines* ^22^. The study is based at Cork University Maternity Hospital, Ireland between 2021 and 2022 and will focus on an international review of clinical practice guidelines. In recognition of the tailored approach that a systematic review of clinical practice guidelines requires, and in recognition of the need for a systematic and reproducible method, a methodological guide for conducting systematic reviews of CPGs was followed during the design of this protocol ^23, 24^.

### Eligibility criteria

The eligibility of CPGs included will be based on the inclusion and exclusion criteria applied to the PICAR framework ^24^, (P: population, clinical indication(s) and condition(s), I: intervention(s), C: comparator(s), comparison(s) and (key) content, A: attributes of eligible CPGs and R: recommendation characteristics) ( Table 1). The search will not contain language restriction and the CPGs will be sought from 2000 to 2021. This systematic review will exclude any recommendations specific to monochorionic pregnancies. For the purpose of this review, CPGs are defined as “systematically developed statements, to assist practitioner and patient decisions about appropriate healthcare for specific clinical circumstances” ^20^.

**Table 1.** PICAR framework (DCDA, dichorionic and diamniotic).

PICAR Framework	Eligibility Criteria
**P**opulation, clinical indication(s), and condition(s).	**Study population** • All guidelines referring to antenatal management of DCDA twin pregnancy, in high income countries. • Humans only **Clinical Investigation** • Antenatal management of DCDA twin pregnancies **Clinical condition** • DCDA twin pregnancy is defined as each fetus having their own separate placenta with its own separate inner membrane (amnion) and outer membrane (chorion). For the purpose of this review, there is a specific focus on antenatal management of DCDA twin pregnancies. • The antenatal period extends from the start of pregnancy to the onset of labour and antenatal care is "essential for protecting the health of women and their unborn children.
**I**nterventions	• Any intervention focusing on the antenatal management of DCDA twin pregnancies
**C**omparator(s), Comparisons, and (key) Content	• Any comparator or comparison • Key clinical practice guideline (CPG) content of interest is DCDA twin pregnancy antenatal management
**A**ttributes of eligible CPGs	**Scope** • Cover any aspect of antenatal care for DCDA twin pregnancies. • Must be published **Language** • Full text CPGs in any language **Year of publication** • 2000 onwards **Developing/ publishing organisation** • CPGs issued or endorsed by national or international scientific societies, professional colleges, charitable organisations, and government organisations will be included. • State/province guidelines will be included e.g. Australia and Canada, where states work independently. **Country of publication** • High income countries (83 countries), as defined by the world bank ^25^. This is due to the large discrepancies in care systems and management of care between high, low and middle-income countries ^26^. **Version** • Current version only **Development process** • Evidence and/or consensus-based **Quality of evidence** • No maximum quality score based on the AGREE II and AGREE-REX instruments are required for eligibility. ALL relevant CPGs will be included in the review. • The quality score will be used to interpret the review findings and will be addressed in the discussion.
**R**ecommendations characteristics and ‘other’ considerations	• Eligible recommendations should be applicable to antenatal care of DCDA twin pregnancies. • Recommendations will be considered if they are located anywhere within the guideline document (e.g. main text, algorithms or tables). • Guidelines addressing any aspect of antenatal management of DCDA twin pregnancies without recommendations are eligible for inclusion

### Information sources

The identification of relevant CPGs will encompass a multi-tiered approach. This search strategy is developed to identify CPGs concerning the antenatal management of DCDA twins, from the year 2000–2021, throughout a broad network of information sources. Systematic bibliographic database searching will identify CPGs published in journals; others will be identified through online searches of grey literature and guideline repositories, or through professional bodies/ organisations. We will search for the relevant professional bodies of all high-income European Union (EU) countries ( as defined by the world bank), Scandinavian countries, and any high-income English-speaking country outside of the EU. If the professional bodies locate their CPGs behind paywalls, do not have CPGs on their website, or if they do not have a website, we will contact them requesting any relevant CPG(s).

A systematic literature search will be performed, covering CPGs published from 2000 to 2021, using the following databases: PubMed, EMBASE, Cumulative Index to Nursing and Allied Health Literature (CINAHL), MEDLINE, Web of Science and OpenGrey. The guideline repositories and professional bodies/organisations are identified in Table 2 and Table 3.

**Table 2.** Guideline repositories.

Guideline repositories	Website
Agency for Healthcare Research and Quality (AHRQ)	https://www.ahrq.gov/
Australian Clinical Practice Guidelines	https://www.clinicalguidelines.gov.au/
Canadian Agency for Drugs and Technology in Health (CADTH)	https://www.cadth.ca/
Canadian Medical Association (CMA) Clinical Practice guidelines database	https://joulecma.ca/cpg/homepage
Emergency Care Research Institute (ECRI)	https://www.ecri.org/
Geneva Foundation for Medical Education and Research. Obstetrics and gynecology guidelines	https://www.gfmer.ch/Guidelines/Obstetrics_gynecology_guidelinesTemplate.htm
Guidelines International Network (GIN)	https://g-i-n.net/
Institute for Clinical Systems Improvement (ICSI)	https://www.icsi.org/guidelines/
Lenus: The Irish Health Repository	https://www.lenus.ie/
National Institute for Health and Care Excellence (NICE)	https://www.nice.org.uk/guidance
Scottish Intercollegiate Guidelines Network (SIGN)	https://www.sign.ac.uk/
Turning Research Into Practice (TRIP) database	https://www.tripdatabase.com/
World Health Organisation (WHO)	https://www.who.int/publications/who-guidelines

**Table 3.** Professional bodies/ organisations.

Organisation	Country/Region
Oesterreichische Gesellschaft fur Gynakologie und Geburtshilfe (OEGGG)	Austria
Royal Belgian Society for Obstetrics and Gynaecology (RBSOG)	Belgium
Hrvatsko drustvo Ginekolozi Opstetricari (HDGO)	Croatia
Cyprus Gynaecological and Obstetrics Society (COGS) (no website)	Cyprus
Czech Gynecological and Obstetrical Society (CGPS)	Czechia
Dansk Selskab for Obstetrik og Gynaekologi (DSOG)	Denmark
Eesti Naistearstide Selts (ENS)	Estonia
Suomen Gynekologiyhdistys (SYG)	Finland
Collège National des Gynécologues et Obstétriciens Français (CNGOF)	France
Deutsche Gesellschaft für Gynäkologie und Geburtshilfe (DGGG)	Germany
The Hellenic Obstetrical and Gynecological Society (HSOG)	Greece
Magyar Nőorvos Társaság (MNT)	Hungary
Institute of Obstetricians and Gynaecologists of the Royal College of Physicians of Ireland (RCPI)	Ireland
Società Italiana di Ginecologia e Ostetricia (SIGO)	Italy
Associazione dei Ginecologi Italiani: oppedalieri, del territorio e liberi professionisti I (AOGOI)	Italy
Societa Italiana di Medicina Perinatale (SIMP)	Italy
Latvian Association of Gynaecologists and Obstetricians	Latvia
Lietuvos akušerių ginekologų draugija (LAGD)	Lithuania
Société Luxembourgeoise de Gynécologie et d’Obstétrique (SLGO)	Luxembourg
Malta College of Obstetricians and Gynaecologists (no website)	Malta
De Nederlandse Vereniging voor Obstetrie en Gynaecologie (NVOG)	The Netherlands
Polskie Towarzystwo Ginekologów I Położników (PTGIN)	Poland
Sociedade Portuguesa de Obstetricia e Medicina Materno-Fetal (SPOMMF)	Portugal
Normas De Orientacao Clinica (NOCS)	Portugal
Servico Nacional De Saude (SNS)	Portugal
Societatea de obstetrica si ginecologie din Romania	Romania
Slovenskej gynekologicko-pôrodníckej spoločnosti (SGPS)	Slovakia
Slovene Association of Trainees in Gynaecologists and Obstetricians	Slovenia
Sociedad Española de Ginecología y Obstetricia (SEGO)	Spain
Svensk förening för Obstetrik och Gynekologi (SFOG)	Sweden
Norsk gynekologisk forening (NFG)	Norway
Royal College of Obstetricians and Gynaecologists (RCOG)	U.K.
The Royal Australian and New Zealand College of Obstetricians and Gynaecologists (RANZCOG)	Australia & New Zealand
American Society of Reproductive Medicine (ASRM)	U.S.
The American College of Obstetricians and Gynaecologists (ACOG)	U.S.
The Society of Obstetricians and Gynaecologists of Canada/Societé des Obstétriciens et Gynécolgues du Canada (SOGC)	Canada
European Board and College of Obstetrics and Gynecology (EBCOG)	Europe
European Association of Perinatal Medicine (EAPM)	Europe
European Society of Human Reproduction and Embryology (ESHRE)	Europe
Society for Maternal-Fetal Medicine (SMFM)	International
World Association of Perinatal Medicine (WAPM)	International
The International Federation of Gynaecology and Obstetrics (FIGO)	International
International Society for Prenatal Diagnosis (ISPD)	International
The International Society of Ultrasound in Obstetrics and Gynecology (ISUOG)	International
Nordic Federation of Societies of Obsteteric and Gynaecology (NFOG)	Scandinavia

### Search strategy

The search strategy was developed with a researcher/midwife (COC), medical librarian (SB), a senior researcher (SL) and a maternal-fetal medicine subspecialist (KOD). The search strategy will include suitable keywords relating to two clinical concepts. The terms will be followed by the truncation symbols * and will be refined using Boolean operators such as AND/OR. Table 4 represents an example of search terms; this will be tailored to each database as appropriate. The search fields will consist of a full text search, on humans only, from 2000–2021. A full text search will be conducted to identify any recommendations specific to DCDA twin pregnancies that may be nested within other guidelines. The search strategy will be developed on a Microsoft word file and will be piloted before the final searches are executed. See *Extended data* ^22^ for a sample search strategy.

**Table 4.** Search strategy.

Concept	Search terms
1: Twin pregnancy	“Twin pregnanc*” OR “Multifetal gestation*” OR “Multifetal pregnanc*” OR “Multiple gestation” OR “Multiple pregnanc*” OR “Fraternal twin*” OR “Dichorionic Diamniotic” OR “Dichorionic twin*” OR Dichorionic OR “Dizygotic twin*” OR Dizygotic OR “Nonidentical twin” OR “Non identical twin”
	AND
2: Clinical guidelines	“Antenatal management” OR “Clinical practice guideline*” OR “Clinical Guideline*” OR Guideline* OR Guidance OR “Best practice” OR Standard* OR “Practice guideline*” OR “Practice bulletin*” OR Management OR Managing

### Study selection

Retrieved records from the database and website ( Table 2 & Table 3) screening will be imported into Mendeley, and duplicates will be removed using the ‘check for duplicates’ function. They will then be imported into Rayyan ^27^ and screened again for duplicates using the ‘detect duplicates’ function. Two independent reviewers (COC and EOC) will screen the records and highlight any guideline meeting the eligibility criteria outlined in Table 1. Records not meeting the eligibility criteria will be excluded. Reviewers will cross examine approx. 10% of the other reviewers’ decisions and any discrepancies identified will be discussed. The PICAR framework will be applied broadly during the initial screening to ensure relevant CPG’s are not excluded ^24^.

The two reviewers (COC and EOC) will then independently screen the full text articles to identify the CPGs eligible for inclusion. The ‘blind function’ will be activated at this point on Rayyan and to qualify for inclusion, a CPG will receive a ‘Yes’ from both reviewers. Any disagreements will be resolved following discussion and consensus. If consensus on eligibility cannot be agreed, a third reviewer with clinical expertise (KOD) will be consulted for input. A PRISMA flow diagram will be created to illustrate the search process. This diagram will map out the process for CPG selection and the number of records identified at each stage based on inclusion and exclusion criteria.

### Data collection

Following identification of relevant CPGs, all associated supplementary documents will be retrieved by COC prior to data extraction and quality assessment. If links to these documents are not provided within the CPG, COC will conduct a search to locate them. All supplementary documents will be verified by EOC to ensure completeness and correct document pairing.

### Data extraction

Data extraction will be completed by one reviewer (COC) and will be independently verified by EOC for accuracy and completeness. A data extraction form will be designed and adapted based on the methodology applied in previous research of CPGs in the field of obstetrics (the RECURRENT study) ^24^. This will be pilot tested to meet the study specified requirements and then applied for the data extraction of the current systematic review ^28^ General information and recommendations for all included CPGs will be extracted using the structured data extraction form in Microsoft Excel. Discrepancies will be resolved through discussion and consensus. If consensus cannot be reached a third reviewer with will be involved (KOD). Any missing information from the CPGs will be recorded as ‘not described’ (ND) in the data extraction form.

The following information will be extracted from the included CPGs:

Clinical guideline

• Title
• Author & year of publication
• Language
• Developing/publishing organisation and/or authors/ funding
• Country/countries of publication
• Description of document provided by the authors (e.g. guideline, clinical practice guideline, practice bulletin etc)
• Version
• Target audience
• Document structure (presence of abstract, scope, recommendations, conclusion, and length of document)
• Type of Guideline (formulated, adapted, updated or revised)
• Topic addressed (Antenatal management of twin pregnancy of broader)
• Development process (evidence based and/or consensus-based)
• Composition of guideline development group
• Peer-reviewed
• System of rating evidence / Quality instrument used during guideline development
• Validity period

Recommendations

• Guideline
• Author
• Year
• Recommendation
• Strength of recommendation
• Quality of evidence
• Recommendation category (specified within guideline)
• Category (defined by reviewers)

### Quality assessment and risk of bias

The quality of the included CPGs will be assessed using the AGREE II and AGREE-REX tools ^29, 30^.

The AGREE II tool is used to assess the CPG development process as a whole ^31^. The criteria encompass 23 items, over six domains and is rated on a 7-point Likert scale. The domains include (i) Scope and purpose (ii) Stakeholder involvement in the development of the guidelines (iii) Rigour of development (iv) Clarity of presentation (v) Applicability (vi) editorial independence in the formulation of recommendations. A noted limitation the AGREE II tool is that scores do not correlate to the quality of the recommendations within the CPG ^32, 33^.

The AGREE-REX tool was designed as a compliment to AGREE II in response to its noted limitations and focuses specifically on the recommendations within the CPG ^31^. AGREE-REX evaluates clinical credibility (are the recommendations evidence based) and ease of guideline implementation. The AGREE-REX instrument will be applied to a group of recommendations within the guidelines rather than the guideline as a whole, as this study will look at the antenatal management of DCDA twin pregnancy (only selected recommendations are of interest). The criteria encompass nine items, over three domains and is rated on a 7-point Likert scale ^29^. The domains include (i) clinical applicability (ii) values and preferences, (iii) implementability.

Two reviewers (COC and EOC) will conduct the quality assessment of the CPGs. The process will be guided by a researcher with methodological experience (SL). Independent appraiser scores will be used to calculate domain scores for AGREE II. AGREE-REX scores will be calculated through consensus of both reviewers, AGREE-REX requires either a consensus score or 5 independent appraisers as per instructions. If consensus cannot be research, a third reviewer (KOD) will assist. The score for each assessment tool will be calculated for each domain independently and we will scale the total as a percentage of the maximum possible score for that domain (as per instructions). To enable fair comparison of the scores, our review will report overall outcomes categorically, using a 5-point Likert scale adapted by previous peer-reviewed systematic reviews ^28, 32, 34^: excellent (81%-100%), good (61%-80%), average (41%-60%), fair (21%-40%) and poor (0%- 20%).

### Data synthesis

As this will be a narrative systematic review, data synthesis and reporting of results will involve a descriptive approach. We will describe the quality appraisal using the AGREE tools scoring, critically reflect on the content within the CPGs, examine any potential shortcomings and establish international comparisons between both the CPG and their recommendations. Recommendations will be assigned to categories; each category title will be decided following review of all recommendations within the CPGs.

### Study status

We are currently conducting full text screening for this systematic review. The study protocol, database/ organisation/ guideline repository search and the preliminary screening are completed.

## Conclusion

The aim of this systematic review is to identify, appraise and examine published CPGs, in high income countries for the management of antenatal care in DCDA twin pregnancies. All twin pregnancies are considered high risk because of the associated increased risk of maternal and neonatal morbidity and mortality. Intertwin vascular anastomoses are present in most monochorionic twin placentas, necessitating different medical management to DCDA twin pregnancies. In recognition of the different care pathways associated with chorionicity, this study will focus on the antenatal management of DCDA twins only. Guidelines are needed to improve the quality of care and standardise clinical practice ^35^. Because twin pregnancies are identified as high-risk pregnancies, with a need for consistent clinical care, a systematic review of the CPGs in this field is a useful first step as a prerequisite for establishing the required high level of care.

## Data availability

### Underlying data

No underlying data are associated with this article.

### Extended data

Open Science Framework: A Systematic Review of Clinical Practice Guidelines for the Antenatal Management of Dichorionic Diamniotic Twin Pregnancies. https://doi.org/10.17605/OSF.IO/9YZ82 ^22^.

This project contains the following extended data:

- Supplementary File 2_PubMed_Sample Search Strategy.docx.

### Reporting guidelines

Open Science Framework: PRISMA-P checklist for ‘A protocol for a systematic review of clinical practice guidelines for the antenatal management of dichorionic diamniotic twin pregnancy’. https://doi.org/10.17605/OSF.IO/9YZ82 ^22^.

Data are available under the terms of the Creative Commons Zero "No rights reserved" data waiver (CC0 1.0 Public domain dedication).

## References

[1] National Institute for Health and Care Excellence: Multiple pregnancy: twin and triplet pregnancies Quality Standard: (QS46). 2019. Reference Source

[2] Lewi L : Monochorionic diamniotic twins: What do I tell the prospective parents? *Prenat Diagn.* 2020;40(7):766–75. 10.1002/pd.5705 32279339

[3] Khalil A O’Brien P : Challenges of twin pregnancy. *Br J Midwifery.* 2007;15(5):266–72.

[4] Bortolus R Parazzini F Chatenoud L : The epidemiology of multiple births. *Human Reproduction Update.* 1999;5(2):179–87. 10.1093/humupd/5.2.179 10336022

[5] Khalil A Liu B : Controversies in the management of twin pregnancy. *Ultrasound in Obstetrics & Gynecology.* 2021;57(6):888–902. 10.1002/uog.22181 32799348

[6] Luke B Brown MB : Contemporary risks of maternal morbidity and adverse outcomes with increasing maternal age and plurality. *Fertil Steril.* 2007;88(2):283–93. 10.1016/j.fertnstert.2006.11.008 17258214 PMC1955760

[7] Narang K Szymanski LM : Multiple Gestations and Hypertensive Disorders of Pregnancy: What Do We Know? *Curr Hypertens Rep.* 2020;23(1):1. 10.1007/s11906-020-01107-4 33210199

[8] Russo FM Pozzi E Pelizzoni F : Stillbirths in singletons, dichorionic and monochorionic twins: a comparison of risks and causes. *Eur J Obstet Gynecol Reprod Biol.* 2013;170(1):131–6. 10.1016/j.ejogrb.2013.06.014 23830966

[9] Ortibus E Lopriore E Deprest J : The pregnancy and long-term neurodevelopmental outcome of monochorionic diamniotic twin gestations: a multicenter prospective cohort study from the first trimester onward. *Am J Obstet Gynecol.* 2009;200(5):494.e1-494.e8. 10.1016/j.ajog.2009.01.048 19375567

[10] O’Farrell I Manning E Corcoran P : Perinatal Mortality in Ireland Annual Report 2017.National Perinatal Epidemiology Centre. 2019. Reference Source

[11] Herruzo AJ Martínez L Biel E : Perinatal morbidity and mortality in twin pregnancies. *Int J Gynaecol Obstet.* 1991;36(1):17–22. 10.1016/0020-7292(91)90172-2 1683296

[12] Draper ES Gallimore ID Kurinczuk JJ : MBRRACE-UK 2019 Perinatal Confidential Enquiry Stillbirths and neonatal deaths in twin pregnancies.The Infant Mortality and Morbidity Studies, Department of Health Sciences, University of Leicester; 2019.

[13] Carroll SGM Tyfield L Reeve L : Is zygosity or chorionicity the main determinant of fetal outcome in twin pregnancies? *Am J Obstet Gynecol.* 2005;193(3 Pt 1):757–61. 10.1016/j.ajog.2005.01.024 16150271

[14] Dubé J Dodds L Armson BA : Does chorionicity or zygosity predict adverse perinatal outcomes in twins? *Am J Obstet Gynecol.* 2002;186(3):579–83. 10.1067/mob.2002.121721 11904627

[15] Khalil A Rodgers M Baschat A : ISUOG Practice Guidelines: role of ultrasound in twin pregnancy. *Ultrasound Obstet Gynecol.* 2016;47(2):247–63. 10.1002/uog.15821 26577371

[16] Hankins GVD Saade GR : Factors influencing twins and zygosity. *Paediatr Perinat Epidemiol.* 2005;19(Suppl 1):8–9. 10.1111/j.1365-3016.2005.00609.x 15670115

[17] Lewi L Deprest J : Management of twin pregnancies: where do we go from here? *Ultrasound Obstet Gynecol.* 2013;41(6):601–4. 10.1002/uog.12502 23712884

[18] Institute of Medicine (US) Committee on Standards for Developing Trustworthy Clinical Practice Guidelines: Clinical Practice Guidelines We Can Trust.Graham R, Mancher M, Wolman DM, Greenfield S, Steinberg E, editors. *Clinical Practice Guidelines We Can Trust*. Washington, D.C.: National Academies Press; 2011. 10.17226/13058 24983061

[19] Boltin D Lambregts DMJ Jones F : UEG framework for the development of high-quality clinical guidelines. *United European Gastroenterol J.* 2020;8(8):851–64. 10.1177/2050640620950854 32878577 PMC7707865

[20] National Clinical Effectiveness Committee, Department of Health Ireland: How to develop a National Clinical Guideline. 2019. Reference Source

[21] Moher D Shamseer L Clarke M : Preferred reporting items for systematic review and meta-analysis protocols (PRISMA-P) 2015 statement. *Syst Rev.* 2015;4(1):1. 10.1186/2046-4053-4-1 25554246 PMC4320440

[22] O'Connor C leitao s O'Donoghue K : A Systematic Review of Clinical Practice Guidelines for the Antenatal Management of Dichorionic Diamniotic Twin Pregnancies. 2021. 10.17605/OSF.IO/9YZ82 PMC10182673 37179347

[23] Hennessy M Dennehy R Meaney S : A protocol for a systematic review of clinical practice guidelines for recurrent miscarriage [version 3; peer review: 3 approved, 1 approved with reservations]. *HRB Open Res.* 2020;3:12. 10.12688/hrbopenres.13024.3 33005862 PMC7477641

[24] Johnston A Kelly SE Hsieh SC : Systematic reviews of clinical practice guidelines: a methodological guide. *J Clin Epidemiol.* 2019;108:64–76. 10.1016/j.jclinepi.2018.11.030 30529647

[25] World Bank: World Bank Country and Lending Groups. 2020. Reference Source

[26] Goldenberg RL McClure EM Saleem S : Improving pregnancy outcomes in low- and middle-income countries. *Reprod Health.* 2018;15(Suppl 1):88. 10.1186/s12978-018-0524-5 29945628 PMC6019988

[27] Ouzzani M Hammady H Fedorowicz Z : Rayyan-a web and mobile app for systematic reviews. *Syst Rev.* 2016;5(1):210. 10.1186/s13643-016-0384-4 27919275 PMC5139140

[28] Hennessy M Dennehy R Meaney S : Clinical practice guidelines for recurrent miscarriage in high-income countries: a systematic review. *Reprod Biomed Online.* 2021;42(6):1146–71. 10.1016/j.rbmo.2021.02.014 33895080

[29] AGREE-REX Research Team: The Appraisal of Guidelines Research & Evaluation-Recommendation EXcellence (AGREE-REX) [Electronic version]. 2019. Reference Source

[30] Brouwers MC Kho ME Browman GP : AGREE II: advancing guideline development, reporting and evaluation in health care. *CMAJ.* 2010;182(18):E839–42. 10.1503/cmaj.090449 20603348 PMC3001530

[31] Florez ID Brouwers MC Kerkvliet K : Assessment of the quality of recommendations from 161 clinical practice guidelines using the Appraisal of Guidelines for Research and Evaluation-Recommendations Excellence (AGREE-REX) instrument shows there is room for improvement. *Implement Sci.* 2020;15(1):79. 10.1186/s13012-020-01036-5 32948216 PMC7501649

[32] Daley B Hitman G Fenton N : Assessment of the methodological quality of local clinical practice guidelines on the identification and management of gestational diabetes. *BMJ Open.* 2019;9(6):e027285. 10.1136/bmjopen-2018-027285 31201189 PMC6576117

[33] Brouwers MC Spithoff K Lavis J : What to do with all the AGREEs? The AGREE portfolio of tools to support the guideline enterprise. *J Clin Epidemiol.* 2020;125:191–7. 10.1016/j.jclinepi.2020.05.025 32473992

[34] Eady EA Layton AM Sprakel J : AGREE II assessments of recent acne treatment guidelines: how well do they reveal trustworthiness as defined by the U.S. Institute of Medicine criteria? *Br J Dermatol.* 2017;177(6):1716–25. 10.1111/bjd.15777 28667760

[35] Kredo T Bernhardsson S Machingaidze S : Guide to clinical practice guidelines: The current state of play. *Int J Qual Health Care.* 2016;28(1):122–8. 10.1093/intqhc/mzv115 26796486 PMC4767049

