# Supplementary material for: A protocol for a systematic review of clinical practice guidelines for the antenatal management of dichorionic diamniotic twin pregnancy
Source: HRB Open Res. 2021 Oct 22;4:115. [Version 1] doi: 10.12688/hrbopenres.13418.1 (PMC11170067; doi:10.12688/hrbopenres.13418.1)
Supplement: Peer review history

# Reviewer report: Reviewer response for version 1

**DOI:** 10.21956/hrbopenres.14621.r39881

**Published:** 2024-06-12

**Authors:** Steffen Ernesto Kristensen

**Affiliations:** 1 University of Copenhagen, Copenhagen, Denmark

Dear Authors,

 Congratulations on your great efforts and systematic approach in developing this highly relevant protocol for a systematic review of clinical practice guidelines (CPGs) regarding the antenatal management of dichorionic twin pregnancies. The planned assessment of included CPGs using the AGREE-II and AGREE REX tools is a major strength and will significantly enhance the potential for publication in a high-impact peer-reviewed international journal.

 Following are a few comments, and suggestions for minor revision.

**General Comments:**

Search Date Limitation:

 As the current work is a protocol, could you please clarify why the search is limited to 2021? It is likely that multiple CPGs have undergone revisions since 2021, which might render the results outdated. Please explain the rationale behind this decision.

Language Restrictions:

 How do the authors plan to address the “no language restriction” policy? Considering the difficulty of reading medical writing in various languages, it would be beneficial to elaborate on this and preferably include specific details in the protocol regarding how translations will be managed.

Focus Areas:

 While it is presumed that multiple CPGs will cover similar aspects of antenatal management for dichorionic diamniotic (DCDA) twin pregnancies, it would be helpful to include a rough list of the intended areas of focus, particularly those of most relevance to the topic.

**Introduction:**

Typographical Error:

 There is a typo in the description of chorionicity and amnionicity: “monochorionic monoamniotic diamniotic” should be corrected to “monochorionic monoamniotic” to align with the following text.

Nomenclature:

 It is recommended to use the conventional nomenclature “dichorionic diamniotic” instead of “dichorionic and diamniotic” for clarity and consistency.

**Table 4: Search Strategy**

 Although the supplied search strategy is an example, it would be beneficial to include abbreviations such as “DCDA” and terms like “DC twin*” which are commonly used in the literature.

 Overall, this protocol presents a robust framework for conducting a systematic review on this important topic. Addressing the above points will further strengthen the protocol and ensure its comprehensive nature.

Is the study design appropriate for the research question?

Yes

Is the rationale for, and objectives of, the study clearly described?

Yes

Are sufficient details of the methods provided to allow replication by others?

Yes

Are the datasets clearly presented in a useable and accessible format?

Not applicable

Reviewer Expertise:

I specialize in clinical work within obstetrics and fetal medicine, with a primary research focus and PhD on multifetal pregnancies, particularly twin pregnancies. I have contributed to the development and revision of multiple national guidelines within the fields of obstetrics and fetal medicine. My expertise includes conducting systematic reviews, as well as performing advanced literature searches and evaluations.

I confirm that I have read this submission and believe that I have an appropriate level of expertise to confirm that it is of an acceptable scientific standard.

# Reviewer report: Reviewer response for version 1

**DOI:** 10.21956/hrbopenres.14621.r31490

**Published:** 2022-02-21

**Authors:** Yasser Amer

**Affiliations:** 1 Pediatrics Department, King Khalid University Hospital, King Saud University Medical City, Riyadh, Saudi Arabia; 2 Guidelines International Network, Perth, Scotland, UK; 3 Clinical Practice Guidelines and Quality Research Unit, Quality Management Department, King Saud University Medical City, Riyadh, Saudi Arabia; 4 Alexandria Center for Evidence-Based Clinical Practice Guidelines, Alexandria University, Alexandria, Egypt

The authors have selected a high risk and priority condition, antenatal management of dichorionic diamniotic twin pregnancy, to conduct a systematic review (SR) of published guidelines using the golden standard for quality assessment of guidelines: the AGREE II Instrument in addition to its new complementary tool: AGREE-REX. They registered their protocol in PROSPERO as well.

 The aim of the study and its methodology section are both well-written, clear, and detailed.

 Systematic reviews and quality appraisals of clinical guidelines are among the invaluable resources that can inform the decision-making of clinicians in general and for obstetricians in particular in this article.

 Using highly cited and validated tools like AGREE II and AGREE-REX is an added value to this work (as recommended by the EQUATOR Network). Moreover, the results may inform guideline development or adaptation for the same health topic that is one of the care gaps in MCH.

 I would recommend adding a statistical analysis in the form of an inter-rater analysis (K statistics) and graphical representation of the 23-items scores (1-7 Likert scale) and the 6 domain scores in addition to the 2 overall assessments (%) of AGREE II in addition to the 3 domains of AGREE-REX to compare and detect and address any discrepancies in the ratings during the quality assessments of CPGs.

Is the study design appropriate for the research question?

Yes

Is the rationale for, and objectives of, the study clearly described?

Yes

Are sufficient details of the methods provided to allow replication by others?

Yes

Are the datasets clearly presented in a useable and accessible format?

Not applicable

Reviewer Expertise:

Clinical Practice Guidelines' review, evaluation, adaptation, development, and implementation. Pediatrics and Child healthcare. Healthcare Quality and Safety. Healthcare Informatics.

I confirm that I have read this submission and believe that I have an appropriate level of expertise to confirm that it is of an acceptable scientific standard.
